# Schizophrenia in the Netherlands: Continuity of Care with Better Quality of Care for Less Medical Costs

**DOI:** 10.1371/journal.pone.0157150

**Published:** 2016-06-08

**Authors:** Arnold van der Lee, Lieuwe de Haan, Aartjan Beekman

**Affiliations:** 1 Kenniscentrum, Zilveren Kruis Achmea, Leusden, The Netherlands; 2 Department of Psychiatry, VUmc University Medical Center and GGZIngeest, Amsterdam, The Netherlands; 3 Department of Psychiatry, Academic Medical Centre, UvA, Amsterdam, The Netherlands; University of Kwazulu-Natal, SOUTH AFRICA

## Abstract

**Background:**

Patients with schizophrenia need continuous elective medical care which includes psychiatric treatment, antipsychotic medication and somatic health care. The objective of this study is to assess whether continuous elective psychiatric is associated with less health care costs due to less inpatient treatment.

**Methods:**

Data concerning antipsychotic medication and psychiatric and somatic health care of patients with schizophrenia in the claims data of Agis Health Insurance were collected over 2008–2011 in the Netherlands. Included were 7,392 patients under 70 years of age with schizophrenia in 2008, insured during the whole period. We assessed the relationship between continuous elective psychiatric care and the outcome measures: acute treatment events, psychiatric hospitalization, somatic care and health care costs.

**Results:**

Continuous elective psychiatric care was accessed by 73% of the patients during the entire three year follow-up period. These patients received mostly outpatient care and accessed more somatic care, at a total cost of €36,485 in three years, than those without continuous care. In the groups accessing fewer or no years of elective care 34%-68% had inpatient care and acute treatment events, while accessing less somatic care at average total costs of medical care from €33,284 to €64,509.

**Conclusions:**

Continuous elective mental and somatic care for 73% of the patients with schizophrenia showed better quality of care at lower costs. Providing continuous elective care to the remaining patients may improve health while reducing acute illness episodes.

## Introduction

In the Netherlands, as in the rest of the world, rising costs of health care have led to critical reappraisals of the organization and costs of health care. In patients with chronic and severe health conditions, it may be better, both in terms of health maintenance for the patient and in terms of containing health care costs, to invest in continuity of appropriate elective care, thereby avoiding disruptive and expensive episodic treatment.

Schizophrenia is a serious mental illness and for most of the patients it is a chronic disease [1]. Many patients also suffer from somatic comorbidities, especially cardiovascular diseases [2–6]. Patients live 15–25 years shorter mostly because of somatic diseases, but also because of suicide [7,8]. Adherence to treatment is problematic. Discontinuity of treatment is associated with psychotic relapse, hospitalization and increased mortality rate [9–14].

Medical care for patients with schizophrenia consists of psychiatric treatment, psychosocial intervention, medication and somatic care. The aim is to improve symptoms, increase quality of life and functioning and to prevent psychotic relapse, acute treatment events and psychiatric hospitalization. Continuity of care is important to achieve these goals [15–26].

To ensure continuity of care for chronic diseases as schizophrenia evidence-based planned care arrangements, such as collaborative care and assertive community treatment have been developed [27–31]. Comparison of programs between countries is difficult because of differences in health care systems, the financing of those systems and because target populations differ. Financial barriers, inadequate treatment or not using evidence-based practices are mentioned as reasons for suboptimal care [32–37]. These differences between countries may be an explanation for the finding that the effectiveness in reduction of hospital admissions by assertive community treatment for severe mental illness in the United States of America could not be replicated in the United Kingdom or in the Netherlands [38–39]. Other studies show that evidence-based programs, as (F)ACT and other types of collaborative care result in better continuity of care and for instance better social functioning of patients with schizophrenia [40–53].

Randomized controlled trials are important to gain insight in optimal care but are mostly based on small numbers of patients. The generalizability of these studies to daily practice often remains unknown. Naturalistic studies on large computerized claims data provide insight in the actual care for large numbers of patients. Most prominent weakness of these naturalistic studies is bias by indication. The latter will be smaller in a country with well-developed health care and easy access to that care. Therefore large claims databases can complement experimental studies and provide information about continuity, quality, and the costs of care.

The health care system for mental and somatic problems in the Netherlands is well developed with easy access for patients. The aim is to provide integrated health care with a well-developed primary care system. In the Netherlands the term elective psychiatric treatment is used for planned comprehensive psychiatric care including medication, psychotherapy, work rehabilitation, family involvement and hospitalization when appropriate [54]. The elective psychiatric care for schizophrenia is performed by Functional ACT teams. The acute psychiatric care is organized separately and includes emergency ambulatory care, acute home visits, care in police stations and reference to a psychiatric hospital when necessary. Inpatient care is defined as psychiatric treatment during hospitalization.

Somatic care is defined as all the health care not provided by the specialized psychiatric care as covered by the Dutch Healthcare Act. The main categories of somatic care are medical care provided by general practitioners, medical specialists and obstetricians, district nursing, hospitalization, medications, medical aids, services provided by therapists like physical therapists, ambulance transport [55].

For patients with schizophrenia the general practitioner does not provide the psychiatric care, because they need specialized care [56]. The general practitioner takes care of all the non-psychiatric primary care together with the other health care providers. Both psychiatric and somatic care are fully provided and are covered by the Dutch Healthcare Act [55,57–59].

Every inhabitant in the country is mandatorily insured with premiums and subsidies that make the health insurance affordable for everybody. The healthcare insurers in the Netherlands have to accept every inhabitant by law and every inhabitant can change every year to another health insurer.

The Dutch health care insurers process and pay the claims for the mental and somatic health care. They have detailed information about their insured and the care that is given.

The combination of easy access to well-developed health care and availability of data about that care for patients with schizophrenia in the Netherlands provides a unique situation compared with other countries to study the distribution and costs of medical care for these patients.

The objective of this study is to assess how many patients with schizophrenia received continuous care in 2009–2011 and whether continuous care is associated with characteristics of quality of care and with less health care costs due to less inpatient treatment.

We evaluated (i) which percentage of patients with schizophrenia received continuous elective psychiatric care and (ii) whether continuous elective psychiatric care is related to less acute treatment events, less inpatient care and more somatic care and (iii) whether this is associated with health cost savings over time.

## Methods

### Study design and patient selection

Computerized claims data of a Dutch Health Insurer (Agis) were collected. All insures covered by Agis were included. Prescription data of antipsychotics and mental and somatic health care use of patients with schizophrenia over 2008–2011 were analyzed in a retrospective cohort study. All patients in the Agis population with the diagnosis of schizophrenia in 2008 were selected. Their health care use in the follow-up period of 2009–2011 was analyzed.

Agis was a main health insurance company in the Netherlands in 2008–2011 and provides health coverage for about 1.2 million residents in the central more urbanized part of the country. The Agis population was somewhat older, had relatively more women and a higher percentage of minorities than the Dutch population^,^ but were otherwise representative for the Dutch population [60].

**Fig 1:**
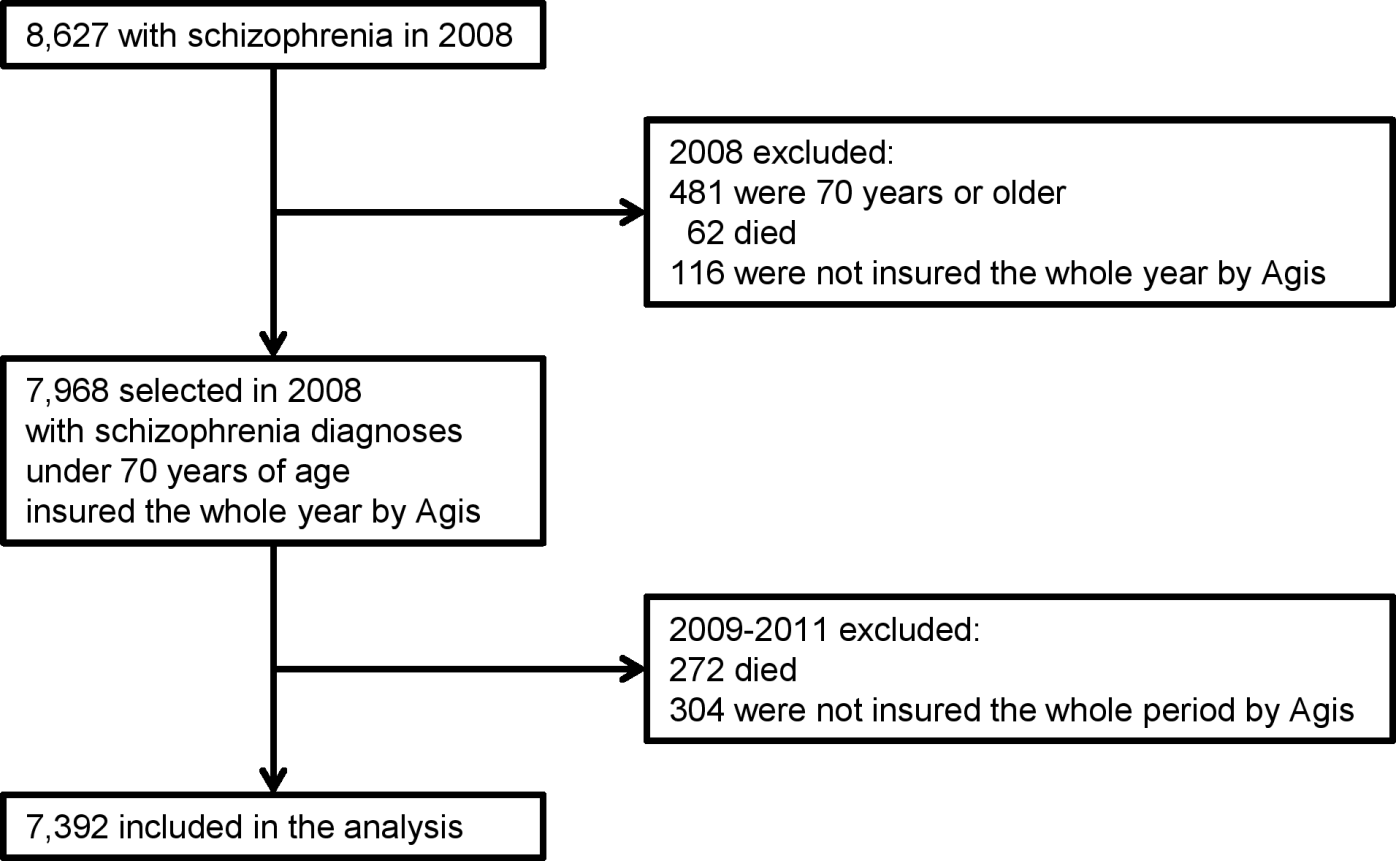
flowchart of the participants in the analysis.

In 2008 there were 8,627 patients who had claims with the diagnosis schizophrenia (Fig 1). 481 (5.6%) patients of 70 years or older were excluded because they differ considerably in their healthcare use and costs because of comorbidity in older adults. In our study the number of older patients is still small, however because of population aging the group may probably increase [61]. From the 8,146 patients left, 62 (0.8%) died in 2008 and 116 (1.3%) others were not insured during the whole year and were excluded. This left 7,968 patients younger than 70 years with schizophrenia who were insured at Agis during the whole year 2008.

In the follow-up period 2009–2011 272 (3.4%) of the 7,968 patients died and 304 (3.8%) were not insured by Agis for the whole period and were excluded. Therefore the study sample consists of 7,392 patients under 70 years of age with a diagnosis of schizophrenia in 2008, who were insured during the whole period 2008–2011 by Agis.

### Data source: Dutch computerized claims data

The Dutch health care insurers process and pay the claims for both mental and somatic health care. The National Care Authority regulates the claims process that is mandatory for both health care providers and insurers. The insurer thoroughly checks the claims. Because of this procedure the Dutch health care insurers have detailed information about their insured population and the care that is given. In this study no data were used that could identify individual patients. Therefore, according to prevailing law in the Netherlands, informed consent or approval of a Medical Ethical Committee was not necessary.

From the Agis dataset the inpatient and outpatient mental care, acute treatment events, medication information, and cost of mental and somatic care over 2009–2011 were used in this study. The database contains all health care from all health care providers.

#### Information from claims

The information about the diagnoses is limited. In case of mental health care only the main groups of diagnoses of DSM-IV are used. For the main diagnosis schizophrenia spectrum disorders the DSM-IV codes included are: 291.3, 291.5, 292.11, 292.12, 293.81, 293.82, 295.10, 295.20, 295.30, 295.40, 295.60, 295.70, 295.90, 297.1, 297.3, 298.8, 298.9. More detailed codes are not provided. Only the diagnosis that is the main focus of the treatment is chosen by the provider and registered on the claim. A patient with schizophrenia can be treated for schizophrenia and also for another diagnosis e.g. depression. If depression is the main focus of the treatment that diagnosis will be registered on the claim. For short-term treatment no diagnosis has to be provided.

If the treatment is for an acute treatment event or inpatient treatment, this type of treatment will be registered on the claim.

The information about outpatient medication is very detailed. It includes the name, price and also the Defined Daily Doses (DDD) of every medication. Information concerning inpatient medication is not available, because the costs are included in the price of the inpatient care.

### Measures

To answer the first question concerning continuity of elective psychiatric care, outpatient psychiatric treatment and the use of antipsychotic medication was analyzed.

Data of antipsychotic medication and outpatient treatment were aggregated per calendar year. A follow-up year of continuous elective psychiatric care is defined as a year with outpatient elective psychiatric care and or the prescription of over 180 DDD’s of antipsychotic medication. The number of follow-up years of elective psychiatric care in 2009–2011 was calculated. The group of patients with three years of elective psychiatric care will be called the “continuous care group”, the group without elective psychiatric care, one year or two years of elective psychiatric care will be called respectively the “no treatment group”, the “one year treatment group” and the” two years treatment group”.

The second question concerns the association between continuous elective psychiatric care and less acute treatment events, less inpatient care and more somatic care. Therefore we assessed: the percentage of patients who had one or more acute treatment events, based on claims which indicated acute care; the percentage of patients with any inpatient care and the average amount of inpatient care per patient, in terms of costs of inpatient care; and the average costs of somatic care per patient. The amount of inpatient care is reflected by its costs. The costs are based on the number of days of hospitalization, therefore more days of hospitalization result in higher costs. The outcome effects were calculated over the follow-up period 2009–2011.

To answer the third question, about health cost savings, costs of psychiatric care and somatic care were calculated over the follow-up period. The total cost of medical care is the sum of the cost of psychiatric and somatic care, covered by the Dutch Health Care act in the follow-up years 2009–2011.

The cost of psychiatric care was calculated as the average cost of the psychiatric care using average national prices. Inpatient treatment is priced based on the number of days in the clinic. Outpatient treatment is based on a price per minute of treatment. The amount of psychiatric care is calculated as the sum of costs of out- and inpatient treatment plus the costs of antipsychotic medication. The costs of somatic care were defined as all other medical care including medication. Therefore it includes also costs of non-antipsychotic medication.

### Analysis

To answer the first question about the continuity of care, four groups of patients were formed based on the number of follow-up years of continuous elective psychiatric care. Every patient in the cohort could have 0–3 years of continuous elective psychiatric care over the follow-up years 2009–2011. The patients with 3 years of continuous elective psychiatric care are considered to have been provided with continuous medical care

The second research question about the putative effects of the continuity of elective psychiatric care was answered by calculating how many patients had acute treatment events or inpatient care and the amount of inpatient care.

To answer the third research question costs of psychiatric care and somatic care were used. The total cost of medical care is the sum of the cost of all psychiatric and somatic care.

The weighted mean effect sizes (Cohen’s *d* with 95% confidence intervals) were calculated between the group with three years of elective psychiatric care and the three other groups of patients. Cohen’s *d* reflects mean differences between treatment groups in standard deviation units, with estimates of 0.2, 0.5, and 0.8 indicating small, medium, and large effects, respectively [62].

### Results

#### Age and Gender

**Fig 2:**
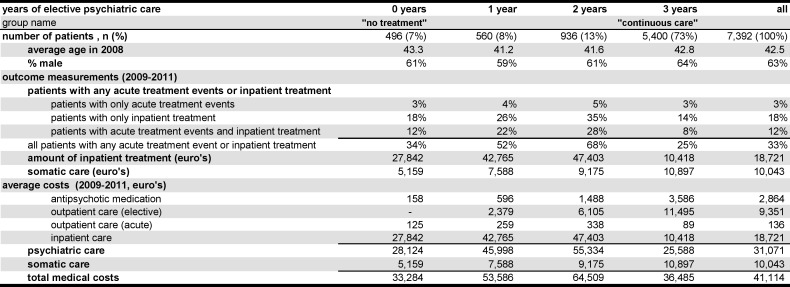
Results by subgroup of years of elective psychiatric care over 2009–2011.

The average age of the cohort was 42.5 (Fig 2). The age differences between the four treatment groups were small. The average age of the subgroups in 2008 ranged from 41.2 to 43.3.

The percentage of males in the cohort was 63% and varied across the subgroups from 59% to 64%.

#### Continuity of care: number of patients in the four treatment groups

The first question is about continuity of elective psychiatric care. The group with all the three years of continuous elective psychiatric care was the largest group with 5,400 patients (73%) of the 7,392 patients in the cohort (Fig 2). There were 936 (13%) of patients with two years of elective psychiatric care, 560 (8%) patients with one year and 496 (7%) had no follow-up years of elective psychiatric care.

#### Outcome measurements: acute treatment events, inpatient care and somatic care

The relation between the number of years of continuous elective psychiatric care and the outcome measures acute treatment events and inpatient care is the subject of the second research question.

33% of the patients had acute treatment events or inpatient treatment in 2009–2011 (Fig 2). The continuous care group with three years of treatment showed least of these outcomes, 25% of the patients had any of those treatments. The groups with less years of treatment suffered more acute treatment events or inpatient treatment with 34% in the no treatment group, 52% in the one year group and 68% in the two year treatment group. A small percentage of patients had only acute treatment events (3%–5%). More patients had only inpatient treatment (14%–35%). And 8%-28% had both types of non-elective treatment. The three years treatment group consistently demonstrated the lowest percentages, followed by the 0 years, 1 year and 2 years treatment groups.

The amount of inpatient care is shown by the cost of inpatient care. The average cost in the whole cohort was €18,721 over the three follow-up years. The continuous care group had, with €10,418, only 55% of the total average costs. The no treatment group had 1.5 times the average costs followed by the one year treatment (2.3 times) and two years treatment groups (2.5 times).

The amount of somatic care demonstrated a strong positive relation with the number of years of elective psychiatric care. The average costs of the whole cohort over the whole follow-up period for somatic care and non-antipsychotic medication together were €10,043. The no treatment group had the least costs with €5,159, followed by the group with one treatment year, then the two year treatment group and €10,897 for the continuous care group.

**Fig 3:**
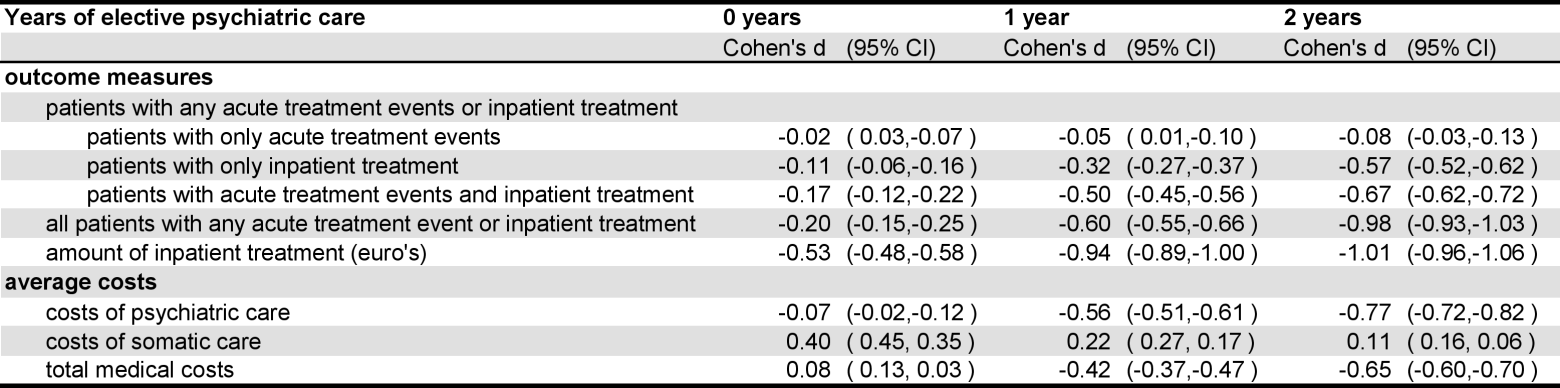
continuous care group with 3 years of elective psychiatric care versus other groups: effect sizes (Cohen’s d with 95% confidence intervals) in the follow up period 2009–2011.

The impression of clinical relevance was shown by the effect sizes between the continuous care group and the other groups (Fig 3). The effect sizes for all patients with any acute treatment event or inpatient treatment between the continuous care group and the one and two years of treatment groups were medium to large. The effect sizes for the amount of inpatient care between the continuous care group and the other three groups were medium to large.

#### Cost of medical care

Whether continuous elective psychiatric care is associated with cost savings over time is the third research question.

The costs of elective psychiatric care did rise as expected, with the number of years of elective psychiatric care (Fig 2). The no treatment group had only some costs of antipsychotic medicine for patients with at most 180 DDD’s per year. The highest cost was found in the continuous care group with €3,586 for antipsychotic medication and €11,495 for elective outpatient care.

The costs of non-elective psychiatric care (acute outpatient care plus inpatient care) were lowest for the continuous care group (€10,507). Much higher were the costs of non-elective psychiatric care in the one year group (€43,024) and the two years group (€47,741).

The total of the costs of psychiatric care ranged from €25,588 from the continuous care group, to €55,334 from the two years group.

The somatic costs, as described in the previous paragraph demonstrated a strong positive relation with the number of years of elective psychiatric treatment and varied from €5,159 to €10,897.

The total costs of medical care were lowest in the no treatment group with €33,284 per patient, closely followed by the continuous care group, much higher in the one year treatment group and the highest total costs were found in the two year treatment group with €64,509.

The effect sizes for costs of psychiatric care between the continuous care group and the one and two years treatment group were medium to large and there was almost no effect for the no treatment group (Fig 3). The costs of somatic care showed a reverse pattern of effect sizes. The total costs showed medium effects between the continuous care group and the one year and two years treatment groups.

## Discussion

Our primary objective was to test whether continuity of elective care is associated with less acute treatment events, inpatient care and less costs of health care for patients with schizophrenia. Less acute treatment events and less inpatient care are indications of better quality of care [15,34,36,37]. We think that our data are appropriate for this goal since all patients with schizophrenia have access to health care in the Netherlands without financial barriers. We found that indeed the majority of patients (73%) are provided with continuous elective care during follow-up and that this was associated with less acute treatment events, less inpatient care and more somatic care. These results are interesting for other countries with reported (financial) barriers or fragmented health care [30,32,33].

We found substantial differences in costs and quality of care between the group with continuity of elective care during the whole three year follow-up period and those without continuous elective care. The patients in the continuous care group received the most elective outpatient psychiatric care and antipsychotic medication, and also the most somatic care. The quality of care was indicated by the low level of acute treatment events or inpatient care, in 25% of the patients, while the costs of inpatient care was the lowest of all groups at €10,043. The other groups did receive less elective psychiatric and somatic care and had more unfavorable outcomes in 34%-68% of the patients while the costs of inpatient care ranged from €27,842 to €47,403. The continuous care group with three years of elective care had the combination of lowest psychiatric care and highest somatic care costs with a total of €36,485, just above the €33,284 for the no treatment group. The two year treatment group demonstrated the highest medical costs with €64,509, followed by the one year group with €53,586.

The high level of continuous care and the substantial differences in hospital admissions in the continuous care group and the other groups in our study compares very favorably to the reported quality health care in the United States [30,33]. These differences in quality of health care may explain why the reduction of hospital admissions by assertive community treatment in the United States could not be replicated in the United Kingdom or the Netherlands [38,39]. The potential for reduction in hospital admissions in the Netherlands might be very small compared to the potential in the United States.

The group without years of elective psychiatric care probably contained more patients who avoided elective psychiatric care till they could not avoid acute treatment or inpatient treatment any longer. In this no treatment group there may be a proportion of patients who had their first psychosis in 2008 followed by several years without need of psychiatric care.

Patients with increasing severity of their symptoms will have higher chance to get acute treatment or inpatient care. Patients with less illness severity may be less likely to seek treatment [35,63,64]. Under elective treatment increasing symptoms severity might be diagnosed earlier. This probably results in remission of symptoms before acute treatment events or inpatient care arises.

The results of our study should be interpreted in the light of several limitations. First, we do not know what caused the differences in care between subgroups. Were the patients less willing to comply with treatment, did they receive psychiatric care in the primary health care, or was the care less well organized? In other studies drop-out of care was associated with more severe symptoms, male gender, lower social status and diminished insight. Therefore, the association we found between continuity of care and less acute treatment events and inpatient care may have been driven by patient characteristics. However, total drop out of care may not be the most important factor associated with acute treatment events and inpatient care since these outcomes were most pronounced in the group who received two years elective care. Therefore, relatively modest interruptions of continuity of care may be associated with worse outcome and higher health care costs.

Second, detailed information concerning other DSM-IV diagnoses or inpatient medication was not available in the dataset we used. Therefore we were not able to study associations between co-morbid psychiatric disorders and outcome and costs.

Third, the group that stayed with Agis insurance during follow-up may be different from those that changed. However, the loss of patients because of change to another insurer is limited. In 2008 only 116 (1.3%) were not insured that whole year by Agis. In 2009–2011 304 (3.8%) patients out of 7,968 patients were not insured by Agis for the whole period.

Fourth, the groups are compared on three outcomes in a naturalistic study: (1) acute treatment, (2) inpatient treatment and (3) costs of psychiatric and somatic care. These outcomes are a consequence of psychiatric and medical comorbidities. Therefore we cannot use the comorbidities as control variables.

Large claims data provide additional insights compared with trials, especially about the actual care for large groups in a population. There are only a few examples of research on claims data. Most of them focus on one aspect of healthcare for patients with schizophrenia, for instance the use of antipsychotics, costs, family services, or continuity of care [18,22,35,61,64–69]. One study examines the relation between continuity of care and increased mortality [10]. In our study we focused on the relationship between continuity of care and quality of care.

Strengths of our study are the reliability of the data. Claims data from health insurers in the Netherlands are comprehensive and include both the mental health care and the somatic health care from all health care providers. Correct data are important for provider and insurer. Moreover, the claims process is well regulated and controlled by the National Care Authority. This ensures the quality of the data.

Continuous elective psychiatric care was given to 73% of the schizophrenia patients, who had less acute treatment events and less inpatient care, more somatic care and less healthcare costs than those with one or two years of elective psychiatric care. Ensuring continuous psychiatric care may achieve better treatment outcome for less costs. There is room for improvement by providing more patients with continuous elective medical care. The financial potential for investing is shown in our data. If successful in changing substandard care to continuous care, outcome may be improved and cost benefits may be up to €10,000 per year per patient.
